# The Role and Therapeutic Potential of the Integrated Stress Response in Amyotrophic Lateral Sclerosis

**DOI:** 10.3390/ijms23147823

**Published:** 2022-07-15

**Authors:** Elías Marlin, Cristina Viu-Idocin, Montserrat Arrasate, Tomás Aragón

**Affiliations:** 1 Neuroscience Program, Center for Applied Medical Research (CIMA), University of Navarra, 31008 Pamplona, Spain; emarlin@alumni.unav.es; 2 Gene Therapy and Regulation of Gene Expression Program, Center for Applied Medical Research (CIMA), University of Navarra, 31008 Pamplona, Spain; 3 School of Medicine, University of Navarra, 31008 Pamplona, Spain; 4 Neuroscience Department, Navarra Institute for Health Research (IdiSNA), University of Navarra, 31008 Pamplona, Spain; 5 School of Sciences, University of Navarra, 31008 Pamplona, Spain; cviu@alumni.unav.es

**Keywords:** amyotrophic lateral sclerosis, integrated stress response, translation, uORFs-containing mRNAs, ALS experimental models, RNA-binding proteins, ALS clinical trials

## Abstract

In amyotrophic lateral sclerosis (ALS) patients, loss of cellular homeostasis within cortical and spinal cord motor neurons triggers the activation of the integrated stress response (ISR), an intracellular signaling pathway that remodels translation and promotes a gene expression program aimed at coping with stress. Beyond its neuroprotective role, under regimes of chronic or excessive stress, ISR can also promote cell/neuronal death. Given the two-edged sword nature of ISR, many experimental attempts have tried to establish the therapeutic potential of ISR enhancement or inhibition in ALS. This review discusses the complex interplay between ISR and disease progression in different models of ALS, as well as the opportunities and limitations of ISR modulation in the hard quest to find an effective therapy for ALS.

## 1. The Unmet Need for Effective Amyotrophic Lateral Sclerosis Therapies

Amyotrophic Lateral Sclerosis (ALS) is a neurodegenerative disease whose progression results from the death of the upper (motor cortex and brain stem) and lower (anterior horn of the spinal cord (SC)) motor neurons (MNs), controlling voluntary movements. Even if ALS is considered a rare pathology, it represents the most common type of motor neuronal disease (MND). ALS symptomatology is characterized by progressive muscle stiffness, twitches, and atrophy, difficulty speaking and swallowing, paralysis, and respiratory failure that eventually leads to death. Although juvenile cases exist, disease onset is typically after 50 years. There is a clear gender-based predisposition to ALS, with men being more prone to developing the disease. Based on the development of motor symptoms, patients can be classified as slow or fast progressors. Patients are also classified according to the location of the first MNs affected, which are defined as limb (the most common), bulbar (usually fast progressors), and trunk onset cases. Unfortunately, regardless of the onset, most patients die 3–5 years after the diagnosis.

The incidence of ALS is around 1.3–3 cases/100,000 inhabitants per year worldwide. The prevalence is more than 200,000 cases worldwide and is projected to increase by 2040 to more than 375,000 cases globally [1]. ALS represents a high economic burden to patients, families, and sanitary systems worldwide [2,3] being one of the most expensive neurological diseases per patient across the globe [3]. Unfortunately, ALS is still a fatal disease. Treatments for ALS are mostly palliative; at the moment, Riluzole (a drug that reduces glutamate excitotoxicity) is used for ALS treatment, extending by 3–6 months the survival of the patients. Recently, Edaravone (an antioxidant drug used for stroke treatment in Japan [4]) was approved in the USA [5] and some other countries for ALS treatment [6]. However, Edaravone might not be effective for all ALS patients, working only in a specific population [7]. Indeed, the European Medicines Agency declined its approval, asking for extensive data, at which the company responded with a withdrawal of the marketing authorization application [8]. Nevertheless, a new formulation as an oral suspension of Edaravone has just been approved by the U.S. Food and Drug Administration (FDA) [9]. In this dire situation, it is urgent to decipher the pathogenic molecular mechanisms driving ALS and to envisage/develop new strategies to stop or delay disease progression.

## 2. ALS; a Complex Disorder, with Distinct Pathological and Molecular Manifestations

In spite of the many efforts dedicated to understanding and curing ALS since the disease was defined in 1869 [10], the precise molecular mechanisms leading to the death of MNs remain unclear. In 90–95% of the cases, ALS appears as a sporadic (sALS), non-inheritable disease, whereas in 5–10% of the cases, it is caused by single mutations in a distinct set of genes that are sufficient to trigger ALS in an inheritable, familial fashion (known as familial ALS or fALS).

Since the first fALS-causing mutations were identified in 1993 in the gene that encodes the superoxide dismutase 1 (SOD1) protein [11,12], the discovery of fALS mutant genes has been instrumental to developing cellular and animal experimental ALS models as a starting point to deciphering ALS pathogenic mechanisms. In the SOD1 gene, more than 200 fALS-linked mutations [13,14] have been described, being A4V, D90A, and G93A some of the most common pathological alleles. SOD1 mutations represent around 12–20% of fALS cases, even being described in sALS cases (1–2%) [15,16]. The abnormal expansion of hexanucleotide repeats (GGGGCC) (>11 repeats) in the first intron of the chromosome 9 open reading frame 72 (C9orf72) gene accounts for more than 40% of fALS patients. C9orf72 mutations represent the most frequent cause of fALS and are detected in 7% of sALS patients [17,18,19]. Noncanonically, sense and antisense C9orf72 expanded repeats are translated by a non-ATG initiated mechanism known as Repeat Associated Non-AUG (RAN) translation [20], yielding different dipeptide repeats or DPRs (polyGR, polyGP, polyGA, polyPR, polyPA) with different associated toxicity [21,22,23]. Beyond the toxicity of DPRs, the RNA repeats concentrate in intranuclear foci and mediate translation-independent mechanisms of neurotoxicity [24,25,26], raising the question of how much toxicity is associated with the RNA versus the DPRs [27,28]. Other important pathological mutations are those related to TAR DNA-binding protein 43 (TARDBP) (also known as Transactive response DNA-binding protein 43 (TDP43) [29,30] and mutations in Fused in Sarcoma (FUS) [31,32,33]. TDP43 and FUS mutations represent around 4% of fALS cases and 1% of sALS cases. Finally, mutations in genes such as Coiled-coil-helix-coiled-coil-helix domain-containing protein 10 (CHCHD10), Vesicle-associated membrane protein-associated protein B/C (VAPB), Ataxin-2 (ATXN2), Optineurin (OPTN), and Ubiquilin-2 (UBQLN2) account for less than 1% of sALS and fALS cases [13,34,35].

The identification of fALS-causing mutations and the development of fALS experimental models have paved the way to identifying altered mechanisms underlying neuronal death. Except for SOD1-related fALS cases (where SOD1 aggregates are found), all ALS patients (familial and sporadic) share as a pathological hallmark the abnormal aggregation of TDP43 protein. Indeed, mutations in C9orf72, SOD1, TDP43, FUS, OPTN, UBQLN2, and VAPB cause the misfolding and accumulation of these proteins in experimental models. Essential processes like RNA metabolism (C9orf72, FUS, TDP43), endocytosis (C9orf72, SOD1), autophagy (SOD1, C9orf72, OPTN) and apoptosis (VAPB, SOD1) are altered [34,36]. Mutations in SOD1 cause oxidative stress, axonal and mitochondrial dysfunction [36], and CHCHD10 mutations also induce mitochondrial alterations [37]. Finally, changes in neuronal excitability underlie neurotoxic mechanisms in familial and sporadic ALS. Both MN hyperexcitability caused by glutamate excitotoxicity [38] and MN hypoexcitability [39] have been observed to be related to the pathogenesis of ALS in animal models. Therefore, multiple pathological stress mechanisms simultaneously converge, triggering the death of MNs in ALS.

## 3. The Integrated Stress Response (ISR)

The Integrated Stress Response (ISR) is a cellular central signaling network that enables the sensing of different stress situations through four kinases: GCN2 (general control nonderepressible 2), HRI (Heme-regulated inhibitor), PKR (Protein kinase R), and PERK (PKR-like endoplasmic reticulum kinase). The activation of these kinases induces a translational and transcriptional remodeling program aimed to restore homeostasis in the cell [40]. 

**Figure 1:**
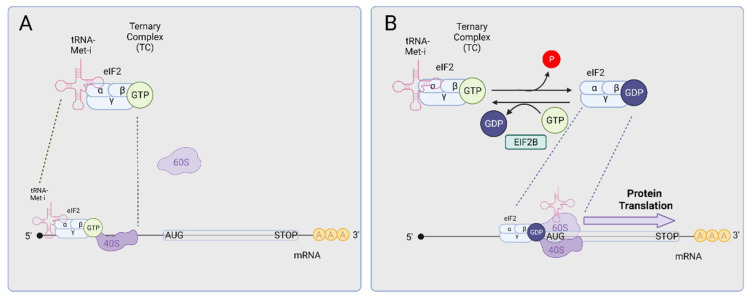
Role of the Ternary Complex (TC) in translation initiation. (**A**) The TC (formed by the three eIF2 subunits (α, β, and γ), a tRNA-Met-i, and GTP) assembles with the 40S ribosomal subunit and scans the 5′ untranslated mRNA regions for AUG initiator codons. (**B**) Once an AUG initiation codon is found, GTP is hydrolyzed to GDP, driving the disruption of the TC, the assembly of the 60S ribosome subunit and the translocation of the tRNA-Met-i into the P-site of the ribosome. This process leads to general protein translation. To initiate new rounds of translation, the eIF2 complex has to be recycled by exchanging GDP for GTP, a process facilitated by the translation initiation factor eIF2B. Created with BioRender.com.

The control of protein synthesis is essential for any cell type under normal or stress conditions. In the case of neurons, localized translation of mRNAs at synaptic terminals determines the efficiency of synaptic transmission and plays a key role in the maintenance of the central nervous system (CNS) circuitry [41]. Most of this control is exerted at the level of translation initiation. One of the limiting steps of translation initiation is the recycling of the ternary complex (TC). The TC is composed of (1) eukaryotic initiation factor 2 (eIF2) which in turn is formed by three subunits (alpha, beta, and gamma), (2) the initiator methionyl transfer RNA (tRNA-Met-i), and (3) guanosine triphosphate (GTP). The TC is assembled into the 40S small ribosomal subunit that scans the 5′ untranslated region (5′ UTR) of mRNAs until reaching AUG initiator codons (Figure 1A). As this preinitiation complex poises on the AUG, GTP hydrolysis drives the disruption of the TC, the assembly of the 60S subunit of the ribosome, the translocation of the tRNA-Met-i into the P-site of the ribosome, and the release of eIF2 bound to GDP. To recycle the eIF2 complex and initiate new rounds of translation, the eIF2-bound GDP moiety must be exchanged by GTP, a process that is facilitated by the translation initiation factor eIF2B (Figure 1B). 

**Figure 2:**
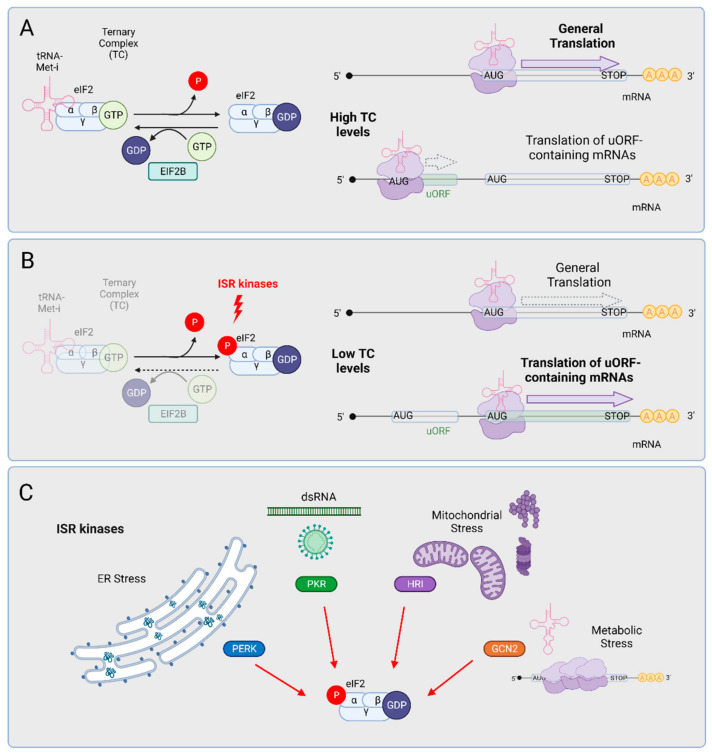
ISR kinases reprogram cellular translation by regulating TC levels through eIF2α phosphorylation. (**A**) After translation initiation, eIF2B recycles the eIF2 complex by exchanging GDP by GTP and favoring high TC levels. Under this situation, the translation of most mRNAs (general translation) is promoted and on the contrary, uORFs-containing mRNAs translation is non-favored. (**B**) Under stress conditions, the ISR kinases phosphorylate the alpha subunit of eIF2, blocking the eIF2B-mediated GDP/GTP exchange. As a consequence, the recycling of the eIF2 complex is impeded and TC levels are low. In this case, general protein translation is inhibited but the translation of uORFs-containing mRNAs is favored. (**C**) Each one of the four ISR kinases is activated by a specific type of stress. Endoplasmic Reticulum (ER) stress caused by misfolded proteins activates PERK, whereas endogenous or viral double-stranded RNAs (dsRNAs) activate PKR. Cytosolic protein aggregation, and proteasome and mitochondrial dysfunction activate HRI. Finally, metabolic stress (such as amino acid starvation or ribosomal stalling) promotes GCN2 activation. Importantly, the activation of all kinases converges on eIF2α subunit phosphorylation, causing a gene remodeling program that relies upon uORFs-containing mRNAs privileged translation. Created with BioRender.com.

Since the TC is a limiting component of the translation machinery, its recycling is essential to ensure that most mRNAs are properly translated (Figure 2). As an exception to this “general translation” rule, the translation of the main open reading frame of mRNAs bearing small upstream open reading frames (5′ uORFs) at their 5′ UTRs is inhibited (Figure 2A). The ISR provides a physiological control of TC levels. In response to a wide variety of stresses, four independent stress sensing kinases phosphorylate the alpha subunit of eIF2 at serine in position 51 (Figure 2B); such phosphorylation promotes the stable association of eIF2 and eIF2B and inhibits eIF2B-mediated GTP exchange. This inhibition reduces TC levels in the cell, leading to two main consequences: (1) the inhibition of general protein synthesis and (2) the enhanced translation of 5′ uORF-containing mRNAs. The best-known paradigm of this type of transcript is the mRNA encoding the activating transcription factor 4 (ATF4) which, as we will discuss below, could play a critical role in ALS pathology. Beyond ATF4, a broad set of transcripts encoding key regulators of neuronal functions also include 5′ uORFs [42]. From a cellular perspective, the inhibition of translation promotes the formation of stress granules (SGs), which are microscopically visible assemblies that form through liquid-liquid phase separation. SGs are composed of translationally arrested mRNAs and RNA-binding proteins, as well as some translation factors (reviewed by English, Green, and Moon, 2022 [43]). SGs form rapidly upon eIF2alpha (eIF2a) phosphorylation and cluster over time; when translation rates are restored, SGs are dynamically “dissolved”. The unique dynamic properties of SGs rely on intrinsically disordered domains present in many of the RNA-binding proteins that assemble in the granules.

Each one of the ISR kinases is activated by a specific type of stress, but all of them signal through eIF2a phosphorylation and determine the same output (Figure 2C); for this reason, this pathway is known as the integrated stress response. Among the four ISR kinases (GCN2, HRI, PKR, and PERK), PERK has been proposed to trigger the pathway in sALS and in fALS models (see below). Still, every ISR kinase has been involved in neuronal regulation, and the different stresses that activate them concur in ALS MNs and glia.

### 3.1. GCN2

GCN2 was identified as a sensor of amino acid starvation. Through its histidyl tRNA synthetase-like domains, GCN2 is able to detect and bind uncharged aminoacyl tRNAs, which leads to the activation of its eIF2a kinase domain. As the transcriptional program elicited by the ISR promotes the biogenesis of new amino acids, and their loading into tRNAs by newly made tRNA synthetases, GCN2 provides the switch to recover homeostasis after starvation. Recently, a new GCN2 activation mechanism has been described, whereby elongating ribosome stalling promotes the association of GCN2 to the ribosomal stalk and its ensuing activation [44]. This unanticipated activity in ribosome quality control may be relevant in the context of neurodegenerative diseases, as it has been established in a model of peripheral neuropathy [45]. To date, the role and therapeutic potential of GCN2 in some of the most prevalent neurodegenerative diseases is controversial. For instance, in an Alzheimer’s disease (AD) mouse model, GCN2 deletion reduced phosphorylated eIF2a (p-eIF2a) levels and improved spatial memory [46] while in a different model, GCN2 deletion seems to be deleterious [47].

### 3.2. HRI

Classically identified as a red blood cell modulator that adjusts globin mRNA translation to heme levels [48,49], new discoveries have illustrated a much broader regulatory role for this kinase, particularly in the central nervous system. Recently, Alvarez-Castelao and coworkers identified HRI as the main sensor and responder to deficiencies in the ubiquitin proteasome degradation. Inhibition of the proteasome allowed the enhanced expression of HRI, leading to the activation of the ISR translational program [50]. In line with this role in proteostasis, aged HRI-deficient mice display an accumulation of protein aggregates and serine 129 alpha-synuclein (aSyn) phosphorylation in the CNS [51], indicating that HRI plays a key role in the clearance of toxic, misfolded proteins and thereby may prevent the loss of proteotoxicity and neurodegeneration. In line with this notion, in a Parkinson’s disease (PD) cellular model based on aSyn overexpression, ISR gene expression is HRI-dependent [52]. 

Moreover, two recent reports identified a new signaling mechanism whereby HRI triggers ISR activation in response to mitochondrial stress. Mitochondrial respiratory dysfunction induces the cleavage of the intermembrane space protein DELE1 (L-DELE1) by the OMA1 protease, releasing a truncated protein fragment (S-DELE1) that then translocates into the cytosol where it associates and induces the activation of HRI and the ensuing ISR [53,54]. As the main driver of ISR homeostatic responses, a recent multi-omics study identified ATF4 as a key transcription factor for the restoration of mitochondrial homeostasis [55]. While this new mechanism provides an unprecedented link between mitochondrial stress and the ISR, ATF4 activation is still observed (with delayed kinetics) in DELE1- and HRI-deficient cells, indicating that ISR kinases other than HRI may provide a functional ISR backup when mitochondria fail; PERK and GCN2 would be good candidate kinases to fulfil this task [56,57,58]. The notion that HRI determines ISR activation featured by two ALS molecular hallmarks, such as cytosolic protein misfolding and mitochondrial failure or dysfunction, is intriguing and hints that—at least in specific types of sALS or fALS—HRI may play a relevant role in disease progression.

### 3.3. PKR

Protein kinase R (PKR) was originally described in vertebrates as a kinase activated by viral double-stranded RNA (dsRNA) [59,60]; however, the repertoire of dsRNA species is much broader and includes intracellular dsRNA, such as dsRNA molecules of mitochondrial origin (mtRNAs) that can form intermolecular dsRNAs [61,62] and are among the most PKR-activating RNAs. PKR is activated by dsRNA by driving its dimerization and trans-auto-phosphorylation [63]. Beyond the direct sensing of dsRNA, PKR can be tuned in a dsRNA-independent mode. PKR is negatively regulated through its interaction with sphingosine kinase 1 (SPHK1) [64] and can also be activated by the PKR-associated protein activator (PACT), or its murine homolog RAX [65,66]. Through these diverse activation mechanisms, PKR responds to a wide range of endogenous stresses such as oxidative stress, changes in calcium concentration, or endoplasmic reticulum (ER) stress. Additionally, cytokines (IFNγ, TNFα, or PDGF) and small molecules have also been described as PKR activators [67,68,69]. The capacity of PKR to detect such a wide array of endogenous and exogenous stimuli is consistent with the possibility that PKR could tune the ISR in MNs and glial cells in ALS.

### 3.4. PERK, the ISR Arm of the UPR

In response to deficiencies in protein folding or lipid membrane composition at the ER, the unfolded protein response (UPR) comprises a set of three independent ER-to-nucleus signaling mechanisms, aimed to restore ER homeostasis (reviewed in Walter and Ron, 2011 [70]). Three transmembrane ER stress sensors, IRE1alpha, ATF6, and PERK, initiate distinct signaling mechanisms under the dysfunctional situation commonly known as ER stress: (1) Upon activation, IRE1alpha catalyzes the non-canonical splicing of a unique mRNA encoding the transcription factor XBP1, which, in turn, enables the translation of XBP1s and the ensuing transcription of its targets. IRE1alpha also cleaves with lower specificity a broader set of transcripts, promoting their decay [71]. (2) ATF6 is an ER transmembrane protein containing a cytosolic transcription factor domain. Under stress, ATF6 translocates into the Golgi apparatus, where it is proteolytically processed to release its transcriptional moiety and enable its transcriptional activity. (3) Finally, the ER transmembrane kinase PERK phosphorylates eIF2a and elicits ISR upon ER stress [70]. PERK activation results from a dimerization/oligomerization orchestrated by the association of misfolded proteins in the ER lumen and the dissociation of the main ER chaperone, BiP. Besides PERK’s capacity to initiate ISR signaling, PERK can phosphorylate the antioxidant transcription factor Nrf2, in a manner that facilitates its translocation into the nucleus and the enhancement of antioxidant transcription [72]. Also, a kinase-independent activity has been identified for, whereby its oligomerization contributes to reorganizing the actin cortical cytoskeleton which, in turn, facilitates extracellular calcium entry [73]. PERK phosphorylation has been observed in post-mortem brain tissue from patients with frontotemporal dementia (FTD), progressive supranuclear palsy (PSP), AD, and PD [74], and is thought to play a key role in a wide range of neurodegenerative disorders, besides ALS (reviewed in Hetz and Saxena, 2017 [74]).

## 4. ISR Activation Is a Molecular Hallmark of ALS

ALS and FTD are probably the neurodegenerative diseases where ISR activation has been best described. In most sALS patients, ISR/UPR markers such as PERK, peIF2α, PDI, BiP, and CHOP have been detected [75,76,77,78,79] together with an abnormal morphology of the ER and the secretory pathway [80,81]. In the case of fALS, ISR/UPR markers were detected either in patient-derived fibroblasts or in neurons differentiated from patient fibroblasts. In the majority of the cases (including mutations in SOD1, CHCHD10, and VABP), patient-derived cultures evidence the loss of protein folding homeostasis and the activation of the UPR, but also alterations in mitochondrial function [82,83,84,85]. Interestingly, this pattern of ISR activation is not the same in all fALS experimental models: in fALS fibroblasts or iPSC-MNs obtained from patients with TDP43 or C9orf72 or FUS mutations, ISR activation is not apparent under control culture conditions but is strongly enhanced under mild ISR-inducing conditions [86,87,88]. In contrast, mitochondrial deficiencies in iPSC-neurons derived from mutant TDP43 patients could be scored even under control conditions [89].

For most of the fALS genes studied, the transgenic expression of mutant, pathogenic alleles (or in a few cases, loss-of-function models) has served to recapitulate their toxicity or specific pathogenic mechanisms. The early discovery of the SOD1 mutations as the first genetic drivers of fALS [12] led to the development of the first ALS transgenic mouse model, the mutant SOD1-based transgenic SOD1^G93A^ mouse. This model faithfully recapitulates many of the main aspects of disease progression [90]. Given the diversity of mutant genes able to cause inheritable ALS, many possible ways to trigger ISR in ALS have been proposed. 

In the probably best-characterized example of a UPR inducing fALS mutant, the interaction of pathogenic SOD1 alleles with Derlin-1 appears to provide the mechanistic explanation to induce the UPR [91]. In sharp contrast with this SOD1-Derlin1 paradigm, most of the fALS mutant proteins/RNAs are not directly localized at the ER or participate in protein folding within the ER, and therefore PERK activation (or UPR-dependent ISR activation) should arise as a secondary effect of neurotoxicity. Even if most studies accept that translational regulation in ALS is determined by PERK, a case-by-case inspection of the ALS models where ISR activation has been documented does not discard that ISR kinases other than PERK could have a major contribution to ALS. For instance, in transgenic SOD1^G93A^ mice, the increase of ISR/UPR targets is attributed to PERK activation, but most of the transcripts upregulated correspond to ATF4 target genes; moreover, non-canonical XBP1 splicing is not significantly increased in these samples [92]. Similarly, translational profiling of MNs from transgenic mice expressing the SOD1 G85R mutation fails to support UPR splicing but shows a robust upregulation of ATF4-dependent target genes [93]. Along with this notion, a neuronal ALS model based on the expression of mutant SOD1 G93A recapitulated the bona fide UPR activation. Still, while ISR inhibition with the downstream inhibitor ISRIB improved neuronal survival, the PERK kinase inhibitor GSK2606414 failed to do so [94]. From these observations, it is tempting to speculate that, given the pleiotropic actions of mutant SOD1 in the ER, cytosol, and mitochondria [95,96], more than one eIF2alpha kinases could act in a concerted manner. In the case of C9orf72, a genome-wide CRISPR Cas9 screen aimed to identify modulators of DPRs toxicity confirmed that DPRs induced robust activation of the ISR but not UPR targets [97]. In agreement with this observation, silencing of GCN2 alleviated DPRs toxicity [97]. Moreover, a recent report demonstrated that in patients with C9orf72 expansions, phospho-PKR levels are increased and contribute to the DPRs pathogenicity [98]. In support of a major role of PERK in ALS neurotoxicity, TDP43 toxicity was alleviated in Drosophila and rat primary cultures through the pharmacological inhibition of PERK [99]. 

The development of mouse ALS models has allowed us to link the activation of the ISR to MNs physiology and to establish its importance through the course of neurodegeneration. In an elegant and seminal work, Saxena et al. demonstrated that fast-fatigable motor neurons, the ones that are more prone to degeneration in a mutant SOD1 mouse, undergo an early and abrupt activation of the ISR that precedes their death in transgenic mutant SOD1 mice. ISR activation was either absent or delayed in slow-resistant MNs [100]. The notion that ISR/UPR markers are detected in the most vulnerable MNs subtype is suggestive that ISR is a fate-defining mechanism in ALS. In iPS-derived MNs, those with larger somas (resembling the vulnerable, fast-fatigable MNs) display a higher UPR amplitude, even if these MNs are derived from healthy cells [82]. Based on this evidence, it is tempting to speculate that ISR activation could be related to the high functional demands of MNs, and fALS mutations could increase the intrinsically high metabolic/functional demands of the MNs or impair the capacity of these MNs to maintain homeostasis. Along with this notion, a nice study from Marin Manuel indicates that, after MN stimulation, there is a physiological burst of ISR (detected by increased peIF2a levels) that is lost when, after repeated stimulation, the ALS neuron becomes hypoexcitable [39]. In other words, a physiological ISR may be necessary for the proper electrophysiological behavior of MNs that may be dysfunctional and pathologically active in ALS MNs.

Together, the ISR activation documented in virtually all fALS experimental models may arise from diverse intracellular stresses, and therefore it can be considered a molecular hallmark of the disease. Still, the amplitude, role, and therapeutic scope of ISR may differ among these models, as we will discuss below.

## 5. Is the ISR a Driver of Neurodegeneration?

Since ISR activation has been typically considered a coping response to stress, ISR has been initially studied as a downstream effect of fALS neurotoxic proteins. However, experimental evidence gathered in many fALS models suggests that, reciprocally, ISR induced after stress episodes can promote the initiation of ALS neurotoxicity. 

In a pharmacological model of chronic ER stress, Medinas and colleagues demonstrated that ER stress favors wild-type SOD1 aggregation and astrocyte activation in the spinal cord of the SOD1^WT^ transgenic mouse, resembling the situation observed in a number of sALS patients [101]. In the case of C9orf72, the intertwining between the ISR and expansion repeats is even closer: the cellular stress induced by RAN translation of C9orf72 induces ISR activation which, in turn, enhances the efficiency of RNA translation, thereby creating a feed-forward loop that contributes to neurodegeneration [102,103]. As we will discuss below, pharmacologically induced ER stress promoted the mislocalization of pathogenic TDP43 mutants into cytosolic aggregates, and this effect was further enhanced by GADD34 inhibition [104]. Following a similar logic, the context of a viral infection is sufficient to promote the stable cytosolic aggregation of mutant FUS, triggering the ALS pathogenic process [105].

The link between ISR and ALS pathogenesis can even occur at a non-cell-autonomous level. Several studies demonstrate that the cerebrospinal fluid from ALS patients can propagate ER stress to naïve cells [106], demonstrating that the propagation of ALS neurodegeneration could be mediated, at least in part, by a non-autonomous transmission of ER stress.

## 6. The Logic for Therapeutic ISR Modulation in Different fALS Models

**Figure 3:**
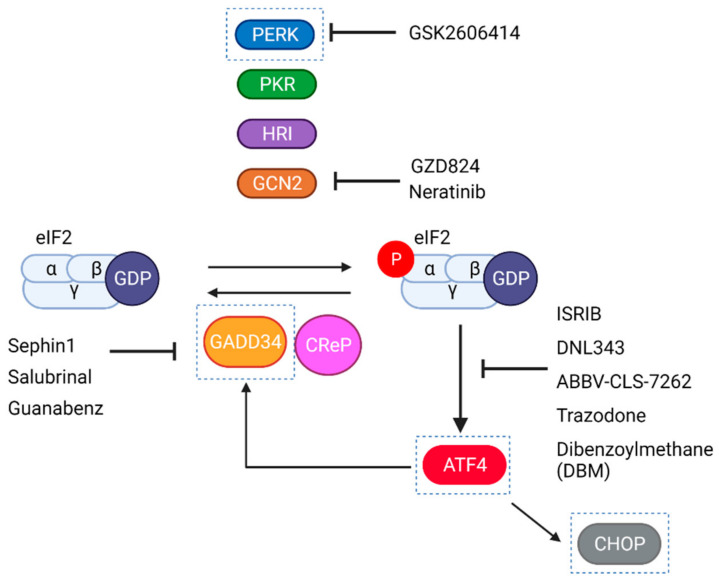
Strategies to modulate the ISR in ALS experimental models. Pharmacologically, (i) ISR inhibition or exacerbation can be achieved by directly tuning either of the four ISR kinases. Most of the studies are focused on the pharmacological inhibition of PERK with the kinase inhibitor GSK2606414 [107]. In the last years, other small molecules have been developed to activate/inhibit other ISR kinases, such as GZD824 or Neratinib, that block GCN2. (ii) The levels of eIF2a phosphorylation can also be tuned by inhibiting phospho-eIF2a dephosphorylation. To that aim, ALS studies have focused on the pharmacological inhibition of GADD34, the stress-induced Protein Phosphatase 1 Regulatory Subunit, required for dephosphorylation of eIF2a. Salubrinal [108], guanabenz [109], or (more recently) sephin1 [110] promote the increased phosphorylation of p-eIF2a by targeting the GADD34-PP1 holoenzyme. Finally, (iii) the ISR can be inhibited by using downstream inhibitors of the pathway, such as ISRIB [111] or its derivatives (DNL343 [112], and ABBV-CLS-7262 [113]). ISRIB, or its derivatives, promotes the dimerization of eIF2B pentamers and allows the recycling of eIF2-GDP into eIF2-GTP even when eIF2a is phosphorylated. Restoration of eIF2B recycling overrules (at least in part) ISR translational regulation. Two FDA-approved drugs, Trazodone and DBM, display ISRIB-like effects under ISR-inducing conditions. Based on their capacity to tune ISR, the repurposing of these compounds has been tested in neurodegenerative models other than ALSs [114]. Beyond these ISR-specific drugs, compounds such as metformin that have a broad effect in metabolism can also affect ISR, [98] and PBA or TUDCA and a TUDCA-derivative (defined as chemical chaperones that mitigate stress) have been also tested in ALS and in patients [115,116,117,118]. In proof of principle studies, the ISR has been modulated using genetic tools/approaches (labeled with dashed blue rectangles to indicate); for instance, the genetic elimination of PERK or the generation of a truncated, inactive version of GADD34 have been used to reduce or enhance the ISR [119,120]. Also, the elimination of ATF4 and CHOP have been attempted [120,121] together with the overexpression of the ATF4-target gene ATF3 [122] and the removal of the UPR transcription factor XBP1 [76]. Finally, the intervention with AAV vectors is a very exciting opportunity to reach the correct cell type, as we can discuss below [123]. Created with BioRender.com.

According to the extensive evidence of ISR activation in ALS, many attempts to establish the therapeutic potential of ISR modulation in ALS have been made in recent years. Through this journey, the different genetic and pharmacological strategies used to inhibit or exacerbate the ISR (see Figure 3) in experimental models of ALS were often successful and led to translation into clinical trials. On the flip side, though, these proof-of-principle independent studies have failed to produce a coherent, unified view on how to develop an efficient therapy for ALS. This inconsistency may be due, at least in part, to the modulation strategy, the molecular target, or the ALS experimental model.

### 6.1. SOD1

The first ALS mouse models generated and the ones that recapitulate the symptoms and disease progression best are based on the transgenic expression of fALS SOD1 mutants. For this reason, most of the studies aimed to explore the therapeutic potential of ISR modulation in these mice. Inspired by the tight link between UPR and ALS, a significant number of these attempts consisted of assessing disease progression in ALS mice lacking specific UPR genes. In 2009, Claudio Hetz and colleagues demonstrated that in a SOD1^G93A^ transgenic mouse lacking neuronal XBP1, the levels of ubiquitinylated, aggregated mutant SOD1 were strongly reduced, disease onset was delayed, and survival times were significantly increased [76]. This recovery in proteostasis and the ensuing alleviation of disease symptoms was facilitated by an increase in autophagic rates that improved the clearance of misfolded proteins [124]. Along with the idea that crippling UPR signaling could be an effective way to stop ALS, SOD1^G86R^ transgenic lacking ATF4 displayed an altered management of SOD1 protein aggregates and delayed disease onset as well as slightly improved survival [121].

In sharp contrast with these studies, studies focused on the genetic or pharmacological modulation of eIF2a phosphorylation/dephosphorylation yielded opposite results: For instance, in SOD1^G85R^ transgenic mice, PERK haploinsufficiency anticipated the early phase of the disease and caused an earlier death of mice [125]. Complementing this view, the genetic inactivation of GADD34 [119] in SOD1^G85R^ transgenic mice delayed disease onset and improved survival [126]. Moreover, a parallel study confirmed that pharmacological inhibition of eIF2a dephosphorylation with guanabenz postponed the early phase of the disease and delayed the death of SOD1^G93A^ transgenic mice [127,128]. Adding to this evidence, treatment of SOD1^G93A^ mice with salubrinal [100] or with an upgraded GADD34 inhibitor, sephin1, sustains a higher ISR tone, prevents the accumulation of insoluble mutSOD1 aggregates, and improves motor function in transgenic mice [110]. Lastly, a strong therapeutic effect was achieved by the neonatal administration of a recombinant adenoassociated virus (AAV) expressing a GADD34-specific shRNA; of note, this gene therapy vector was not effective when administered at the time of disease onset [129].

Considering the main findings and the particularities of each ISR modulation attempt in these studies, the easiest interpretation is that the ISR functions as a pro-survival, coping response induced at the early stage of disease. Unfortunately, the efficacy/reproducibility of some of these works has been questioned. In a recent, comprehensive study, the Popko laboratory used different mouse ALS models based on the transgenic expression of mutant SOD1 alleles, and tried to reproduce the effect of PERK haploinsufficiency, functional GADD34 depletion, or CHOP deletion in disease progression. None of the treatments significantly affected the onset or survival of these animals [120]. Similarly, according to another report, guanabenz accelerates disease symptoms in the SOD1^G93A^ transgenic mice [130]. The discrepancy between these studies hints that, in order to be therapeutically effective, ISR modulation (1) must occur in the proper context or intensity and/or (2) ISR encodes distinct outputs that have to be finely tuned in order to achieve a robust therapeutic effect. Consistent with the second possibility, recent work from our group has demonstrated that the downstream ISR inhibitor ISRIB, decreases significantly the risk of death of SOD1 G93A-expressing neurons. ISRIB tunes neuronal ISR in a unique manner, relieving the translational repression imposed by eIF2a while maintaining (or even increasing) translation of the uORF-containing mRNA, ATF4 [94,131]. From these findings, we propose that the preferential translation of uORF-containing mRNAs encodes a pro-survival activity that, together with the relief of the translational block imposed by eIF2a promotes the survival of ALS cells. According to this model, the exacerbation of ISR may also be neuroprotective by eliciting a robust transcriptional response to stress (provided that general translation is not repressed too strongly). On the other side, total ISR inhibition would eliminate essential pro-survival components and may worsen mutant SOD1-induced pathology.

The notion that uORF-dependent translation can protect neurons under stress and promote survival of ALS MNs is nicely supported by the neuroprotective effect achieved by the transgenic overexpression of the ATF4 target gene, ATF3. In the SOD1^G93A^ mouse, ATF3 transgenic overexpression was sufficient to maintain motor neuron integrity, prevent denervation and muscle atrophy, delay disease onset, and prolong survival [122]. 

### 6.2. C9ORF72

Expanded repeats at the intronic sequence of C9orf72 have been proposed to exert neurotoxicity resulting from (1) the insufficient production of C9ORF72 protein caused by the expansion of intronic sequences, (2) the concentration of C9orf72 repeat-containing sense and antisense RNAs into RNA foci, where RNA binding proteins would be sequestered, and (3) the RAN translation of the intronic sense and antisense RNAs, yielding different types of DPRs. DPRs have been shown to block protein turnover pathways, impair nucleocytoplasmic transport of proteins and cause ER stress. Along with these neurotoxic activities, DPRs induce ISR activation in primary neurons and flies [97,132]. Most likely, C9orf72-elicited ISR results from the disruption of ER proteostasis, as transcriptomic studies from C9-ALS patients show deregulation of UPR gene expression in the frontal cortex and cerebellum [133]. Reciprocally, ISR-inducing stresses promote RAN translation and therefore facilitate the synthesis/accumulation of higher levels of DPRs [102,103,134,135]. In this context, inhibition of ISR puts a brake on the pathogenic process. In transgenic C9orf72 flies, ISRIB or the PERK inhibitor GSK2606414 were strongly neuroprotective [132]. Along with the notion that C9orf72-induced ISR was mediated by the UPR, the ER stress inhibitor tauroursodeoxycholic acid (TUDCA) also reduced neurotoxicity in primary neurons [117]; intriguingly, in the same study, salubrinal also displayed a neuroprotective effect. As most studies consistently support the benefit of ISR inhibition, the identity of the kinase driving ISR in this model is unclear. For instance, in cell cultures challenged with poly-PR (20 repeats), ISRIB was able to decrease cell death; in this model, ISR may be triggered by GCN2 since CRISPR/Cas9 silencing of GCN2 also prevented neuronal death, suggesting that it may be the main driver of ISR [97]. In C9orf72 BAC transgenic mice, PKR activation accounted for the enhanced RAN translation of DPRs and the establishment of behavioral pathogenic changes. The pharmacological inhibition of PKR with the FDA-approved drug metformin [136] reduced DPRs levels and mitigated disease in this animal model [98].

### 6.3. TDP43 and FUS

TDP43 and FUS are shuttling proteins that accumulate in the nucleus. Both factors are RNA-binding proteins containing in their sequences low complexity domains that regulate mRNA metabolism at many steps, including splicing, stability, and translation [137,138]. In the case of FUS also DNA repair [138]. Under ISR-inducing stress conditions, FUS and TDP43, as well as two other fALS-inducing RNA-binding proteins, hnRNPA2/B1 (heterogeneous nuclear ribonucleoprotein A2/B1) and TIA-1, localize into SGs. In sporadic forms of ALS or in response to mutations in either of these proteins, FUS and TDP43 are relocated into the cytoplasm where they form insoluble aggregates [139], and their presence in the nucleus is at least partially depleted. Thus, neurotoxicity of mutant FUS or TDP43 could result from a cytoplasmic gain-of-function toxicity emerging from the cytosolic aggregates or from a nuclear loss-of-function phenotype.

A growing body of evidence supports a complex link between ALS FUS/TDP43 mutant proteins with SG dynamics, translational regulation, and neurotoxic protein aggregation. Mutant FUS has been shown to localize in SGs [140], and also forms aggregates with a protein composition distinct from “standard” SGs [141]. Using humanized mouse models where endogenous FUS was replaced by human wild-type or mutant FUS, ALS/FTD motor and cognitive effects were recapitulated. In this model, mutant FUS localized at axonal foci, causing an early ISR activation that blocked axonal translation without affecting nuclear FUS functions [142], indicating the early activation of ISR could mediate the first steps of pathology. Cytosolic mutant FUS could, as some authors propose, be mediated by UPR activation, although the activation of IRE1 or ATF6 UPR branches is not conclusive [143,144].

In response to transient, ISR-inducing stresses, TDP43 is also recruited to SGs [145,146,147,148]. While the nature of TDP43 incorporation into SGs is reversible (and distinct from the stable, insoluble inclusions that feature advanced pathogenic TDP43 aggregation) it has been proposed that chronic or repeated ISR activation/SGs formation could favour the initiation of pathological TDP43 aggregation [43,104,149,150].

In a comprehensive study in yeast, Drosophila, and neuronal primary cultures, the neurotoxicity of overexpressed TDP43 could be strongly alleviated by the PERK inhibitor GSK2606414 or by ISRIB [99]. Again, steering the ISR in the opposite direction can also convey neuroprotection: The treatment of worms expressing a mutant version of TDP43 with guanabenz or salubrinal also provided a therapeutic benefit that the authors attributed to a decrease in ER stress [99]. 

### 6.4. Clinical Trials with Pharmacological Modulators of the ISR

Some ISR treatments advanced to clinical trials. In a recent clinical trial in which sporadic patients were treated with guanabenz to evaluate safety and efficacy, there was a surprising result that showed that patients with bulbar onset did not progress to a higher stage during the course of the study [151]. However, some patients drop the treatment due to secondary effects of Guanabenz [151]. Nevertheless, some of these drugs have secondary effects in other tissues. GSK2606414 showed some weight loss and mild hyperglycemia in a mouse prion model [152,153] whereas, in ALS patients, guanabenz showed adverse effects such as hypotension, fatigue, and drowsiness that could be related with its alpha-2 adrenergic receptor activity. However, no serious adverse events were observed with respect to the placebo group [151]. For those reasons, clinical trials have evaluated the safety, availability, and tolerability of ISR modulation with sephin1 (IFB-088, that lacks the alpha-2 adrenergic receptor activity and blood pressure lowering adverse effects have not been observed [154]) instead of with guanabenz. Other drugs, such as ISRIB, are not soluble enough to be given to patients (ISRIB) [114]. ISRIB-derived compounds with improved solubility have been developed (such as DNL-343) and tested in healthy volunteers [155,156] that are now progressing to clinical trials in ALS patients [113,157,158]. Finally, a clinical trial to assess the safety and tolerability of Metformin in patients with C9orf72 ALS is ongoing [159] as well as another one with Tradozone (clinical trial phase III for ALS NCT04302870).

## 7. Conclusions and Open Questions

In the quest for effective ALS therapies, different ISR modulation strategies have been tested in different cellular and animal ALS models. Each of these experimental models, with specific limitations and opportunities, has served to establish the curative potential of either ISR inhibition or enhancement. From these studies, ISR targeting compounds have moved into clinical trials and bring new hope to the community of ALS patients and researchers. Surprisingly, at first sight, these studies failed to identify a common approach to alleviate the neurotoxicity of mutant SOD1, TDP43, FUS or C9ORF72, among other proteins. Instead, the emerging view is that the type of ISR intervention should be tailored to the molecular features of each type of ALS. In this complex context that combines hope and uncertainties, we face exciting open questions that remain to be answered:

### 7.1. Should ISR Therapy of ALS Be Personalized?

To date, inhibition of eIF2a dephosphorylation and fine-tuning of ISR with ISRIB derivatives constitute the best options to treat ALS. Since these treatments steer the ISR in opposite directions, either of these strategies could be effective in a subset of fALS and (more importantly) sALS patients but may aggravate the disease in other cases. This heterogeneity in the reaction to ISR modulation may put at risk the success of clinical trials. To circumvent this adverse scenario, the identification of molecular markers that enable the stratification of patient cohorts based on their sensitivity to ISR modulation drugs could give clinical trials a better chance of success.

### 7.2. Is ISR Modulation Therapeutically Effective after Disease Diagnosis?

In mutant SOD1 mouse models, ISR activation is activated in vulnerable MNs before the onset of symptoms and, in most cases, effective ISR modulation was applied before disease onset. Based on this limited evidence, tuning ISR may prevent disease initiation, but not its progression. Thus, it is key to determine if ISR-based therapies could be effective in patients at the stage of disease diagnosis. In that regard, the capacity of some ISR target genes, like FGF21, to limit neuroinflammation [160], suggests that ISR modulation could limit inflammation and disease spreading.

### 7.3. A good Opportunity for ALS Gene Therapy

Translational profiling of the different cell types in the spinal cord of ALS mice revealed that ISR activation occurs selectively in MNs [93], but not in other cell types. Considering the deep role of translational regulation in organismal physiology, the systemic inhibition/stimulation of the ISR could lead to strong metabolic changes or display undesired effects in non-target tissues. For instance, in experimental models of muscle atrophy, ATF4 expression promotes the loss of muscle mass [161]. In the context of ALS neurodegeneration, the modulation of ATF4 in muscle fibers and MNs may require cell-specific tuning to prevent adverse, off-target effects. The development of upgraded adeno-associated vectors that efficiently cross the brain-blood barrier [162] as well as effective methods to deliver therapeutic AAV administration by subpial injection [123] opens up new opportunities to modulate ISR in a cell-specific manner. In this context, recent improvements in methods for gene delivery/modulation have been successfully applied in ALS preclinical models. Silencing of mutant SOD1, C9orf72 or FUS genes in pre-clinical models with cerebrospinal fluid delivered antisense oligonucleotides (ASO) or virally delivered shRNAs have demonstrated the validity of those approaches as a potential therapy in ALS patients [123]. Indeed, different clinical trials are ongoing in ALS patients with ASO targeting SOD1, C9orf72, and FUS transcripts (ClinicalTrials.gov identifiers: NCT04856982, NCT04288856, NCT04931862, and NCT04768972). These state-of-the-art therapeutic tools could also be applied to modulate the ISR.

In spite of the many challenges that the search for a cure for ALS poses, the modulation of the ISR has gathered attention from patients, basic and translational researchers, and biotechnological initiatives. As the first clinical trials with ISR-modulating drugs will deliver (hopefully) good news, what we have learned so far from experimental ALS models will be instrumental in improving and personalizing ISR-based therapies to treat each ALS patient in the future.

## Data Availability

Not applicable.
